# Stereotactic Radiotherapy after Incomplete Transarterial (Chemo-) Embolization (TAE\TACE) versus Exclusive TAE or TACE for Treatment of Inoperable HCC: A Phase III Trial (NCT02323360)

**DOI:** 10.3390/curroncol29110692

**Published:** 2022-11-16

**Authors:** Tiziana Comito, Mauro Loi, Ciro Franzese, Elena Clerici, Davide Franceschini, Marco Badalamenti, Maria Ausilia Teriaca, Lorenza Rimassa, Vittorio Pedicini, Dario Poretti, Luigi Alessandro Solbiati, Guido Torzilli, Roberto Ceriani, Ana Lleo, Alessio Aghemo, Armando Santoro, Marta Scorsetti

**Affiliations:** 1 Radiotherapy and Radiosurgery Department, IRCCS Humanitas Research Hospital, Rozzano, 20089 Milan, Italy; 2 Radiation Oncology Unit, Azienda Ospedaliero Universitaria Careggi, 50134 Florence, Italy; 3 Department of Biomedical Sciences, Humanitas University, Pieve Emanuele, 20072 Milan, Italy; 4 Medical Oncology and Hematology Unit, IRCCS Humanitas Research Hospital, Rozzano, 20089 Milan, Italy; 5 Department of Radiology, IRCCS Humanitas Research Hospital, Rozzano, 20089 Milan, Italy; 6 Division of Hepatobiliary and General Surgery, IRCCS Humanitas Research Hospital, Rozzano, 20089 Milan, Italy; 7 Department of Hepatology, IRCCS Humanitas Research Hospital, Rozzano, 20089 Milan, Italy

**Keywords:** ablative therapy, liver neoplasms, clinical trial, radiotherapy, local treatment, TACE, TAE, SBRT, HCC, BCLC

## Abstract

*Background:* Hepatocellular carcinoma (HCC) is the most frequent liver malignancy and a leading cause of cancer death in the world. In unresectable HCC patients, transcatheter arterial (chemo-) embolization (TAE/TACE) has shown a disease response in 15–55% of cases. Though multiple TAE/TACE courses can be administered in principle, Stereotactic Body Radiotherapy (SBRT) has emerged as an alternative option in the case of local relapse following multiple TAE/TACE courses. *Methods:* This is a single-center, prospective, randomized, controlled, parallel-group superiority trial of SBRT versus standard TAE/TACE for the curative treatment of the intermediate stage of HCC after an incomplete response following TAE/TACE (NCT02323360). The primary endpoint is 1-year local control (LC): 18 events were needed to assess a 45% difference (HR: 0.18) in favor of SBRT. The secondary endpoints are 1-year Progression-Free Survival (PFS), Distant Recurrence-Free Survival (DRFS), Overall Survival (OS) and the incidence of acute and late complications. *Results:* At the time of the final analysis, 40 patients were enrolled, 19 (49%) in the TAE/TACE arm and 21 (51%) in the SBRT arm. The trial was prematurely closed due to slow accrual. The 1- and 2-year LC rates were 57% and 36%. The use of SBRT resulted in superior LC as compared to TAE/TACE rechallenge (median not reached versus 8 months, *p* = 0.0002). PFS was 29% and 16% at 1 and 2 years, respectively. OS was 86% and 62% at 1 year and 2 years, respectively. In the TAE arm, PFS was 13% and 6% at 1 and 2 years, respectively. In the SBRT arm, at 1 and 2 years, PFS was 37% and 21%, respectively. OS at 1 and 2 years was 75% and 64% in the SBRT arm and 95% and 57% in the TACE arm, respectively. No grade >3 toxicity was recorded. *Conclusions:* SBRT is an effective treatment option in patients affected by inoperable HCC experiencing an incomplete response following ≥1 cycle of TAE/TAC.

## 1. Introduction

Hepatocellular carcinoma (HCC) is the most frequent liver malignancy and a leading cause of cancer death in the world [1]. Surgical resection ± liver transplants are curative treatment options for early-stage disease, yielding a 60–80% 5–year survival rate and a recurrence rate of 15–50% at 3 years [2,3]; comparable figures have been reported for other less invasive local ablative options, such as thermal ablation with radiofrequency/microwave (RFA/MWA) [4,5]. However, only 20–40% of patients are eligible for surgical therapy [6,7], while resorting to thermal ablation may be limited by tumor size and topography [8].

Transcatheter arterial embolization (TAE) consists of the selective cannulation of vessels supplying the tumor and occlusion by embolic particles, leading to extensive necrosis in large vascularized HCC. The procedure can be delivered with the concurrent administration of chemotherapy (TACE), although substantial variability in relation to the type of embolic particle, the chemotherapy agent of choice and the treatment regimen has been observed: most interestingly, no clear evidence that TACE is superior to TAE is currently available in terms of either survival [9,10,11] or disease control [12]. TACE is the standard of care for intermediate-stage disease according to the Barcelona Clinic Liver Cancer (BCLC) staging system and may be proposed to early-stage patients unfit for curative surgery [2,3]. The reported response rates to TAE/TACE vary between 15 and 55% of cases [13]. However, while the benefit to survival in comparison to Best Supportive Care (BSC) has been historically reported [14,15], this notion was challenged by a 2011 Cochrane meta-analysis [16]. It has been proposed that a single cycle of TAE/TACE may not be sufficient, and thus, multiple sessions may be needed to consolidate disease control in order to obtain a survival benefit [17,18]. The persistence of a viable tumor documented by contrast-enhanced CT may motivate retreatment; however, it is advised not to repeat the procedure if substantial necrosis is not obtained after two treatment courses [3]. The use of conventionally fractionated radiation therapy has been historically limited by the risk of radiation-induced liver disease [19] despite reports of the superior efficacy of combined TAE/TACE and radiation therapy as compared to exclusive TAE/TACE [20,21]. However, in the last decade, Stereotactic Body Radiotherapy (SBRT), delivering focused ablative radiation doses in a short time interval with the limited irradiation of surrounding healthy tissues, has emerged as a promising treatment option for primary liver malignancies, resulting in high local control rate with a low incidence of radiation-induced liver disease [22]. The aim of this study is to assess the role of SBRT in the multimodality management of unresectable HCC following an insufficient response to the first course of TAE/TACE as compared to TAE/TACE continuation in a phase III prospective trial.

## 2. Materials and Methods

### 2.1. Ethics

This study was registered on ClinicalTrials.gov (accessed on 22 September 2022) (NCT02323360). The trial was conducted according to the Guidelines for Good Clinical Practice, the Declaration of Helsinki, and national regulations. The study protocol was approved by the Institutional Review Board. Informed written consent for entry into the trial was mandatorily obtained prior to randomization. All authors had access to the study data and reviewed and approved the final manuscript.

### 2.2. Patient Eligibility

Adult patients with Karnofsky Performance Status (KPS) >70% were eligible if they were diagnosed with unresectable HCC by histology or non-invasive European Association for the Study of the Liver criteria following prior TAE or TACE with radiologically defined residual disease [17]. Patients also had to be appropriate candidates for locoregional treatment with stage BCLC A-B HCC (without evidence of active extrahepatic disease, including vascular thrombi) and Child–Pugh Class A or B liver disease without existing encephalopathy or ascites. Patients were excluded from the trial in the event of concurrent malignancy, uncontrolled infection, severe anomalies in blood tests, previous abdominal irradiation and Grade ≥3 hemorrhagic complications within 4 weeks of enrollment in the study.

### 2.3. Study Design and Treatment 

**Figure 1:**
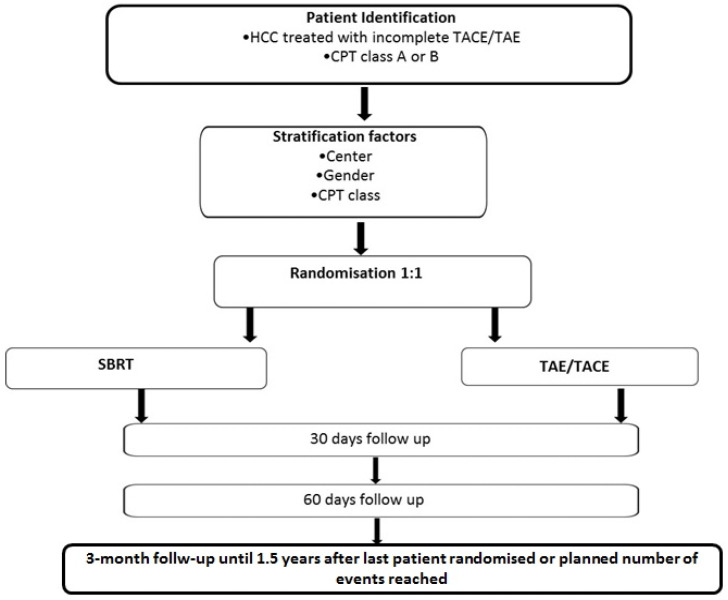
Study Plan. CPT: Child–Pugh–Turcotte. SBRT: Stereotactic Body Radiotherapy. TAE/TACE: Transarterial Embolization/Transarterial Chemo-Embolization.

NCT02323360 was a prospective, single-institution, randomized, controlled, unblinded, parallel-group phase III superiority trial of SBRT versus a second course of TAE/TACE following the incomplete response of unresectable HCC previously treated with one TAE/TACE cycle (Figure 1). Patients with BCLC A-B disease were randomized after the radiological CT-based detection of residual disease (6–8 weeks after TAE/TACE) on a 1:1 basis to receive SBRT or TAE/TACE. A computer-generated minimization program that incorporates a random element was used to ensure that treatment groups were balanced for gender and Child–Pugh class. Patients randomized in the TAE/TACE arm were treated with TAE or conventional TACE according to the first embolization treatment received before the randomization. For TAE, the superselective catheterization of feeding vessels with microcatheters (Renegade High Flow; Boston Scientific, Natick, MA, USA) was obtained to perform subsegmental embolizations. Embolization was performed using small, precise and tightly calibrated microparticles (40 ± 10 and/or 100 ± 25 μm in diameter) with a hydrogel core and a nanothin coating of Polyzene-F (Embozene Color-Advanced Microspheres; CeloNova BioSciences, Newnan, GA, USA). The injection of 40 μm microspheres was performed until blood flow interruption or the administration of 4 mL. In the case of the residual enhancement and/or patency of feeding arteries, 100 μm microparticles were administered. In patients treated with TACE, 5 mL of iodized oil (Lipiodol; Guerbet Japan, Tokyo, Japan) and 50 mg of epirubicin dissolved in 5 mL of non-ionic contrast media were used, followed by particle administration. The procedure was considered completed only when vascular shutdown was confirmed and no feeding vessels to the target tumor were detected at final angiography. In patients randomized to SBRT, a blank and contrast-enhanced 4D-simulated CT was acquired, and, when available, image fusion with magnetic resonance imaging and/or choline positron emission tomography for better target definition was performed. The clinical target volume (CTV) corresponded to the gross tumor volume, as delineated on pretreatment imaging. The planning target volume (PTV) corresponded to the clinical target volume plus a 7–10 mm isotropic expansion. Tumor motion was managed through 4D CT and abdominal compression. SBRT was delivered in 3 to 6 fractions: the dose schedule was adapted on an individual basis in terms of the number of fractions and total delivered dose in order to prioritize with respect to dose–volume constraints for normal tissues, particularly the dose to the residual healthy liver. Patients had baseline contrast-enhanced CT imaging studies of the chest, abdomen and pelvis within 1 month before inclusion to assess the presence of residual HCC following the first TAE/TACE course and within 2 months after the study procedure. Further clinical evaluation was performed every 3 months until 1.5 years after randomization or the progression of the treated site. Relapse before enrollment following the first course of TAE/TACE and treatment failure after the study procedure was defined according to Modified Response Evaluation Criteria In Solid Tumors (mRECIST) criteria for HCC [23]. After radiological progression, any recurrent lesions or distant intrahepatic lesions could receive any treatment according to the clinician’s judgment. Adverse events were summarized by treatment group and graded according to the Common Terminology Criteria For Adverse Events v.5 (CTCAE) for all patients.

### 2.4. Statistical Analysis

All efficacy outcomes were assessed in the intention-to-treat population. Primary (control of local disease, LC) and secondary endpoints (Overall Survival, OS; Distant Recurrence-Free Survival, DRFS; and Progression-Free Survival, PFS) were assessed from the time of treatment to local failure, death from any cause and disease progression at untreated intra-/extrahepatic sites and at any site, respectively. Outcomes were analyzed by the Kaplan–Meier method and compared with the log-rank test. In addition, multivariate analyses using the Cox proportional hazards model were performed to adjust the treatment effect for baseline prognostic factors. The sample size calculation was based on the assumption that at least 50 patients were needed for randomization (25 per arm, 50% randomization rate in both arms) to achieve 80% power to detect an LC Hazard Ratio (HR) of 0.18 (corresponding to a 45% difference at the analysis time, 85% vs. 40% in favor of SBRT versus TAE/TACE [13,17,18,20,21,24,25,26,27,28,29,30,31]) with a 5% two-sided type I error. This corresponded to 18 events following study initiation: as of 23rd September 2019, enrollment was prematurely stopped before planned target accrual was reached (*n* = 40) due to the early achievement of the pre-specified planned number of events. All statistical analyses were performed using MedCalc Version 9.4.

## 3. Results

### 3.1. Patient Characteristics and Treatment

**Figure 2:**
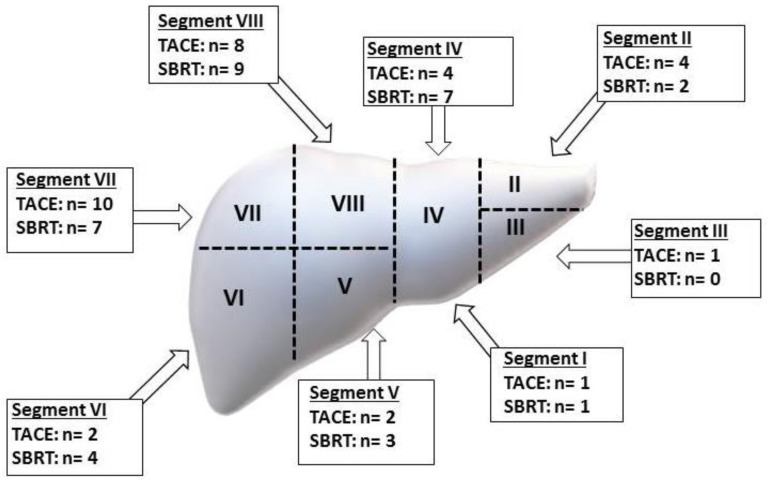
Tumor locations of treated lesions.

From November 2014 to September 2019, a total of 41 patients were included; one patient was subsequently lost before the administration of the study treatment and was excluded from the study. The remaining 40 patients were randomized to SBRT (*n* = 21) or TAE/TACE continuation (*n* = 19): among the latter, 15 patients received bland embolization, and 4 received epirubicin-based TACE. The median follow-up duration was 20 (range 3–56) months. The median age was 75 (range 52–85) years. The median time from diagnosis to randomization was 60 months (range 4–280). The male gender was predominant (*n* = 30, 75%). Hepatitis C virus was the most represented etiology (*n* = 22, 56%), while Hepatitis B virus was found in eight patients (20%); Hepatitis C virus/Hepatitis B virus co-infection was present in three cases. Alcohol abuse was a suspected causative factor in 11 (27%) patients and coexisted with Hepatitis B virus and Hepatitis C virus infection in 1 and 1 patient, respectively. A metabolic etiology was suspected in three (8%) patients. Liver disease was scored Child A and B in 31 (77%) and 9 (23%) patients. The BCLC stage was A and B in 6 (16%) and 34 (84%) patients. The longest median diameter of treated hepatic lesions was 24.8 mm. All patients received at least one prior TACE administration to the target HCC lesion for standard and experimental treatments. Excluding the study procedures, one or more previous locoregional treatments were performed in 48% (*n* = 17) of patients, consisting of RFA, surgery and Percutaneous Ethanol Injection (PEI) in 14 (35%), 8 (20%) and 4 (10%) cases. No patient received a liver transplant. The target lesions were not treated with local therapies other than TAE/TACE before randomization. Between the two arms, the baseline characteristics were well balanced, as confirmed by Fisher’s exact test (Table 1). The locations of treated tumors are summarized in Figure 2. For patients with multiple hepatic nodules, the lesions were targeted separately during TAE/TACE or SBRT. In patients treated with SBRT, the median delivered dose was 60 (30–75) Gy in 6 (3–10) fractions, corresponding to a median Biological Effective Dose of 88 (60–96) Gy^10^.

### 3.2. Local Control

**Figure 3:**
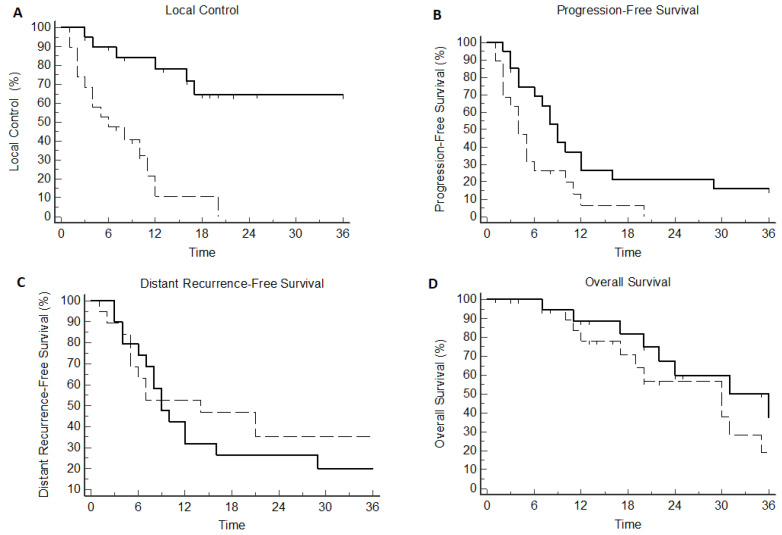
Kaplan–Meier plot according to treatment arm for local control (**A**), Progression-Free Survival (**B**) Distant Recurrence Free Survival (**C**) and Overall Survival (**D**). Solid line: Stereotactic Body Radiotherapy. Dashed line: Transarterial Embolization/Transarterial Chemo-Embolization.

Median LC was 12 months (CI95% 7–20) in the overall population, translating into 1- and 2-year LC rates of 56% and 36%, respectively. The use of SBRT was significantly correlated with superior LC as compared to TAE/TACE (median not reached vs. 8 months, *p* = 0.0002; HR: 0.15 [CI95% 0.04–0.4]), corresponding to a 1-year LC of 84% vs. 23% (Figure 3A and Table 2). No other clinical- or treatment-related variables were correlated with the incidence of local failure.

### 3.3. Progression-Free Survival

Median PFS was 6 (CI95% 4–9) months; 1- and 2-year PFS was 26% and 11%. Patients treated with SBRT experienced significantly longer PFS in comparison with patients treated with TAE/TACE (median 9 versus 4 months, *p* = 0.016; HR: 0.43 [CI95% 0.21–0.87]). In the TAE arm, PFS was 13% and 6% at 1 and 2 years, respectively. In the SBRT arm, 1- and 2-year PFS was 37% and 21%, respectively (Figure 3B and Table 2).

### 3.4. Distant Recurrence-Free Survival

One and two-year DRFS was 76% and 50%, respectively, corresponding to a median DRFS of 24 (CI95% 18–52) months. Median DRFS was 14 months (95% CI 5–21) in the TAE arm and 9 months (95% CI 7–16) in the SBRT arm (*p* = 0.494). No clinical- or treatment-related variables were associated with improved DRFS (Figure 3C and Table 2).

### 3.5. Overall Survival

Median OS was 30 (CI95% 20–36) months, corresponding to a 1-year and 2-year OS of 86% and 62%, respectively. Median OS was 31 months (95% CI 22–53) in the SBRT arm and 30 months (95% CI 17–35) in the TAE/TACE arm (*p* = 0.472). OS at 1 and 2 years was 75% and 64% in the SBRT arm and 95% and 57% in the TACE arm, respectively. In the univariate analysis, OS was significantly impacted by Child–Pugh A liver disease (median 31 versus 17 months, *p* = 0.022; HR 0.22 [CI95% 0.05–0.80]) and prior aggressive locoregional management including surgery and/or RFA/PEI (median 47 versus 22 months, *p* = 0.007; HR 0.28 [CI95% 0.11–0.70]). No correlation was found between the treatment arm and OS (Figure 3D); however, when considering the subsequent use of SBRT following progression in patients initially allocated to TAE/TACE, the integration of SBRT at any time point in the treatment sequence was correlated with improved OS (median 31 versus 19 months, *p* = 0.01; HR 0.21 [CI95% 0.06–0.69]) (Figure S1 Supplementary Materials). In Cox proportional hazard regression, only prior locoregional treatment was significantly correlated with improved survival (*p* = 0.012) (Table 2 and Figure S2 Supplementary Materials).

### 3.6. Patterns of Failure 

At the time of our analysis, local progression occurred in 21 patients following TAE/TACE (*n* = 15) or SBRT (*n* = 6). The pattern of the first failure was local, intrahepatic and extrahepatic in 19, 15 and 3 patients. In the event of progression (any site), 8 patients in the SBRT arm received a further TAE/TACE administration, while 10 patients in the TAE/TACE arm received SBRT. No patients received repeat SBRT after progression. Other treatment options included RFA, surgery, systemic therapy and PEI in 4, 3, 3 and 2 patients, respectively.

### 3.7. Toxicity

The occurrence of toxicity was infrequent (*n* = 5, 13%) and mainly consisted of grade 1–2 nausea and abdominal pain in 4 out of 5 patients (3 in the SBRT arm occurred during radiation treatment and 1 in the TAE/TACE arm occurred within 24 h of the embolization session). Only one patient experienced acute grade 3 sepsis complicated by pleural effusion following TACE. Overall, no grade >3 toxicity was observed in any treatment arm (Table 3).

## 4. Discussion

In patients with intermediate BCLC score (stage B) disease or in other patients unsuitable for surgery due to general conditions, inadequate liver reserve or unfavorable tumor location, the use of TAE/TACE is supported by randomized control trials over supportive care. It has been postulated that one cycle of TACE or TAE may not be sufficient for effective treatment: however, attempts to develop a score for eligibility for repeated TAE/TACE yielded mixed results [24,25]. The use of stereotactic radiotherapy (SBRT) may result in a higher response rate with a reduced incidence of adverse events. SBRT has been proposed as an alternative option [26,27] or used as a complementary treatment after an insufficient response to TAE/TACE [28,29] or in tumors ≥5 cm [30]. We therefore planned a phase III study comparing SBRT with TAE/TACE continuation in patients relapsing after the first course of TAE/TACE. A significant advantage in terms of local control was found in our study in favor of SBRT, meeting the pre-specified objective. Most interestingly, 1-year LC (83%) in patients treated with SBRT in our study is in line with data from the literature, ranging from 77 to 99% in prospective trials [30,31,32,33,34]. Concerning TAE/TACE, the 23% 1-year LC shown in our study is similar to the 12–43% rate observed in a prior series [26,35]. However, while the 1–year PFS rate was significantly higher in the SBRT arm, this effect was probably influenced by the increased local control of treated tumors, as shown by the non-significant difference in the occurrence of distant recurrences. Hence, TAE/TACE did not confer an advantage in terms of disease control outside the target tumor despite the possibly better anatomic coverage of other potential sites of metastatic diffusion. Interestingly, overall survival did not differ between the two treatment arms. However, the study protocol allowed for the administration of the investigational treatment after the primary endpoint was reached. Hence, an influence on the survival of SBRT at any time point in the treatment sequence was shown in the univariate analysis. This was not confirmed by multivariate analysis, where a predominant effect of prior locoregional treatment (excluding SBRT, since previous irradiation was an exclusion criterion) was found, although this may be related to a selection bias favoring fit patients eligible for multiple locoregional treatment sessions. The toxicity profile was mild in both treatment arms. Adverse events consisted of transient gastrointestinal symptoms, not requiring pharmacological intervention, in 13% of patients. Only one patient experienced S. Homini sepsis complicated by pleural effusion and dyspnea, requiring hospital admission and IV antibiotic therapy 2 weeks after TAE. Despite the theoretical risk of liver dysfunction in the SBRT arm, a low incidence of radiation-induced liver disease may be explained by patient selection and the careful application of dose-adapted regimens.

One of the main criticisms of our study is that, despite its phase III design, only 40 patients were included in the trial. Nevertheless, the magnitude of the effect on local control allowed us to ascertain the significant superiority of SBRT, even in a relatively small population according to the statistical plan, reflecting a considerable >45% difference in the primary endpoint. For this reason, accrual was stopped before the enrollment of the planned number of patients when the threshold number of events was reached. While larger multicentric trials are ongoing [36], a confirmation of our results is expected. It may also be speculated that the predominant use of TAE as compared to TACE, in accordance with local institutional practices, may have resulted in inferior response rates. However, our results in the TAE/TACE group are in line with previously published results [26,35], and, while evidence in favor of the inferior activity of bland embolization is lacking, the EASL guidelines support the use of TACE as the preferred treatment modality on the grounds of the better availability of this technique rather than demonstrated superior efficacy [3].

On the other hand, the main limitation of our study is the heterogeneity of the study population, since unresectable HCC eligible for TAE/TACE encompasses different patient subsets with variable lesion sizes, patterns of disease and degrees of underlying liver dysfunction. A non-significant trend toward a higher number of treated lesions in the TAE/TACE group was observed, which may have favored the SBRT group. Moreover, despite the clear inclusion criteria, randomization process and statistical strategies implemented to ensure a good balance between treatment arms, future studies should consider a patient stratification system beyond the BCLC stage to select the best candidates for TACE with or without locoregional ablative therapies. Considering the relatively short follow-up time, we must acknowledge that late toxicities could have been underestimated. Finally, despite the non-significant differences between treatment arms in terms of prior locoregional therapies, the relative weight of each prior intervention (surgery, RFA and PEI) on the outcome cannot be ruled out.

## 5. Conclusions

In this phase III open trial, patients affected by inoperable HCC experiencing an incomplete response following ≥1 cycle of TAE/TACE, SBRT was correlated with significantly higher LC rates as compared to rechallenge with TAE/TACE. This was correlated with extended PFS in the SBRT arm, though no difference was found in the onset of new lesions outside the target tumor. Although no significant OS advantage was found for SBRT over TAE/TACE, an aggressive locoregional schedule may improve outcomes in selected patients.

## Figures and Tables

**Table 1:** Patient- and treatment-related characteristics.

	TAE/TACE (*n* = 19)	SBRT (*n* = 21)	*p*
Gender			
Male	15 (79%)	15 (71%)	0.7
Female	4 (21%)	6 (29%)	
Age (median 75 years, range 52–85)			
<75	9 (47%)	7(33%)	0.5
≥75	10 (53%)	14 (67%)	
Child–Pugh Liver Disease Score			
A	14 (74%)	17 (81%)	0.7
B	5 (26%)	4 (19%)	
Barcelona Staging System (BCLC)			
A	2 (11%)	4 (19%)	0.6
B	17 (89%)	17 (81%)	
HCV infection			
Yes	10 (53%)	12 (57%)	1.0
No	9 (47%)	9 (43%)	
HBV infection			
Yes	4 (21%)	4 (19%)	1.0
No	15 (79%)	17 (81%)	
Alcohol Abuse			
Yes	5 (26%)	6 (29%)	1.0
No	14 (74%)	15 (71%)	
Time from first diagnosis to randomization (median 60 months, range 4–280)			
<60	8 (42%)	11 (52%)	0.5
≥60	11 (58%)	10 (48%)	
Number of treated lesions (median 1, range 1–3)			
1	14 (67%)	9 (48%)	0.12
2	2 (9%)	7 (36%)	
3	5 (24%)	3 (16%)	
Any previous local treatment			
Yes	7 (37%)	12 (57%)	0.2
No	12 (63%)	9 (43%)	

**Table 2:** Statistical analysis assessing the impact of clinical- and treatment-related variables on outcome. SBRT: Stereotactic Body Radiotherapy. TAE/TACE: Transarterial Embolization/Transarterial Chemo-Embolization.

	Local Control	*p*	Progression-Free Survival	*p*	Distant Recurrence-Free Survival	*p*	Overall Survival	*p*
Gender								
Male Female	11 months not reached	0.34	7 months 5 months	0.87	11 months 5 months	0.3	29 months 30 months	0.33
Age (median 75 years, range 52–85)								
<75 ≥75	16 months 11 months	0.94	5 months 7 months	0.45	8 months 9 months	0.59	29 months 30 months	0.99
Child-Pugh Liver Disease Score								
A B	11 months Not reached	0.94	7 months 2 months	0.18	11 months 9 months	0.98	30 months 16 months	**0.027**
Barcelona Staging System (BCLC)								
A B	Not reached 11 months	0.17	7 months 5 months	0.21	8 months 9 months	0.99	30 months 29 months	0.44
HCV infection								
Yes No	11 months 16 months	0.48	7 months 5 months	0.97	20 months 8 months	0.14	30 months 30 months	0.54
HBV infection								
Yes No	11 months 16 months	0.72	5 months 5 months	0.58	9 months 7 months	0.60	29 months 29 months	0.54
Alcohol Abuse								
Yes No	16 months 11 months	0.78	4 months 8 months	0.39	7 months 14 months	0.13	19 months 30 months	0.99
Number of treated lesions (median 1, range 1–3)								
1 >1	12 months 16 months	0.88	7 months 6 months	0.44	10 months 9 months	0.37	31 months 22 months	0.2
Time from first diagnosis to randomization (median 60 months, range 4–280)								
<60 ≥60	11 months 16 months	0.94	4 months 7 months	0.79	7 months 14 months	0.35	29 months 29 months	0.68
Treatment Arm								
TAE/TACE SBRT	5 months not reached	**0.002**	4 months 8 months	**0.035**	14 months 8 months	0.49	29 months 30 months	0.28
Use of SBRT at any time after randomizatrion								
Yes No	N/A	N/A	N/A	N/A	N/A	N/A	30 months 18 months	0.012
Any previous local treatment								
Yes No	not reached 11 months	0.1	5 months 7 months	0.34	9 months 8 months	0.98	not reached 21 months	**0.024**

The numbers in bold indicate *p*-value < 0.05.

**Table 3:** Summary of treatment-related adverse toxicity events. CTCAE: Common Terminology Criteria For Adverse Events. SBRT: Stereotactic Body Radiotherapy. TAE/TACE: Transarterial Embolization/Transarterial Chemo-Embolization.

CTCAE Grade	Nausea		Abdominal Pain		Sepsis	
	SBRT	TAE/TACE	SBRT	TAE/TACE	SBRT	TAE/TACE
1	1 (3%)	0	1 (3%)	1 (3%)	0	0
2	1 (3%)	0	0	0	0	0
3	0	0	0	0	0	1 (3%)
4	0	0	0	0	0	0
5	0	0	0	0	0	0

## Data Availability

Research data are stored in an institutional repository and will be shared upon request to the corresponding author.

## Supplementary Materials

The following supporting information can be downloaded at: https://www.mdpi.com/article/10.3390/curroncol29110692/s1, Figure S1: Kaplan Meier plot for Overall Survival according to use of Stereotactic Body Radiotherapy (SBRT). Solid Line: SBRT at any time point. Dashed Line: No SBRT.; Figure S2: Kaplan Meier plot for Overall Survival according to prior local therapy. Solid Line: use of prior local therapy. Dashed Line: No prior local therapy.
