# Supplementary material for: Stereotactic Radiotherapy after Incomplete Transarterial (Chemo-) Embolization (TAE\TACE) versus Exclusive TAE or TACE for Treatment of Inoperable HCC: A Phase III Trial (NCT02323360)
Source: Curr Oncol. 2022 Nov 16;29(11):8802–13. doi: 10.3390/curroncol29110692 (PMC9689962; doi:10.3390/curroncol29110692)
Supplement: Supplementary file 1 [file curroncol-29-00692-s001.zip › curroncol-2007644-supplementary.pdf]

Supplementarry

Stereotactic Radiotherapy after Incomplete Transarterial (Chemo-) Embolization (TAE\TACE) Versus Exclusive TAE or TACE for Treatment of Inoperable HCC: A Phase III Trial (NCT02323360)

Tiziana Comito [1], Mauro Loi [2], Ciro Franzese [1,3], Elena Clerici [1], Davide Franceschini [1], Marco Badalamenti [1], Maria Ausilia Teriaca [1], Lorenza Rimassa [3,4], Vittorio Pedicini [5], Dario Poretti [5], Luigi Alessandro Solbiati [5], Guido Torzilli [3,6], Roberto Ceriani [7], Ana Lleo [3,7], Alessio Aghemo [3,7], Armando Santoro [3,4] and Marta Scorsetti [1,3]

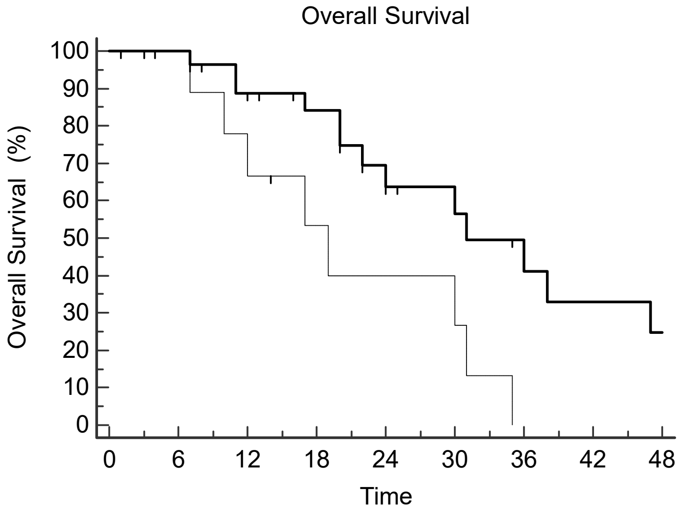

Figure S1. Kaplan Meier plot for Overall Survival according to use of Stereotactic Body Radiotherapy (SBRT). Solid Line: SBRT at any time point. Dashed Line: No SBRT.

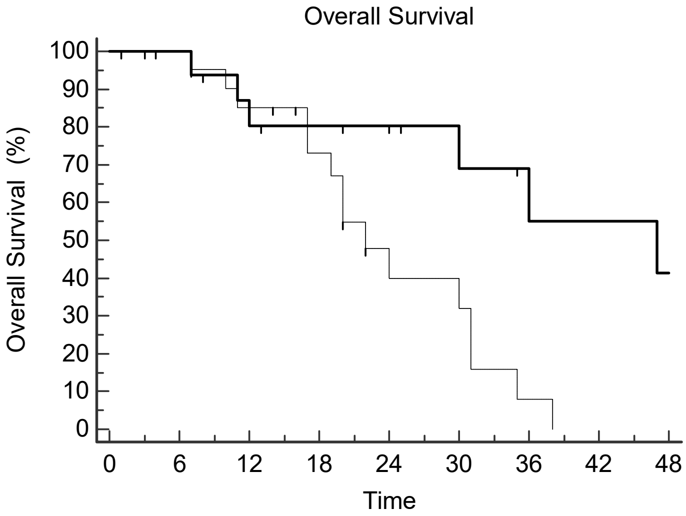

Figure S2. Kaplan Meier plot for Overall Survival according to prior local therapy. Solid Line: use of prior local therapy. Dashed Line: No prior local therapy.
